# Deciphering timing and rates of Central German Chernozem/Phaeozem formation through high resolution single-grain luminescence dating

**DOI:** 10.1038/s41598-023-32005-9

**Published:** 2023-03-23

**Authors:** Hans von Suchodoletz, Mike van Meer, Peter Kühn, Katja Wiedner, Torsten Schunke, Tony Reimann

**Affiliations:** 1 Institute of Geography, Leipzig University, Johannisallee 19a, 04103 Leipzig, Germany; 2 Soil Geography and Landscape Group, Wageningen University, Droevendaalsesteeg 3, 6708 PB Wageningen, The Netherlands; 3 Chair of Soil Science and Geomorphology, Tübingen University, Rümelinstraße 19-23, 72020 Tübingen, Germany; 4 Present Address: Gütegemeinschaft Kompost Ost E. V, Prießener Straße 21, 03253 Doberlug-Kirchhain, Germany; 5 State Office for Monument Preservation and Archaeology of Saxony-Anhalt, Richard-Wagner-Straße 9, 06114 Halle (Saale), Germany; 6 Institute of Geography, University of Cologne, Zülpicher Straße 45, 50923 Cologne, Germany; 7 Institute of Agronomy and Nutritional Sciences, Soil Biogeochemistry, Martin-Luther University Halle-Wittenberg, Halle/Saale, Deutschland

**Keywords:** Environmental impact, Sustainability, Agroecology, Ecosystem services

## Abstract

Chernozems/Phaeozems are important agricultural resources and have been intensively used for millennia. However, their origin and age are still controversial. In Europe, the westernmost widespread Chernozem/Phaeozem area is located in Central Germany. In contrast to other German regions with anthropogenic Chernozems/Phaeozems, their natural origin is suggested in connection with intensive bioturbation. Yet, radiocarbon is unsuitable for decoding Chernozem/Phaeozem formation so this hypothesis remains untested, whereas single-grain luminescence dating allows to discriminate between different soil sub-processes and formation phases. We applied single-grain feldspar luminescence to a Central German Chernozem that was buried during the Bronze Age and subsequently protected from pedogenic processes. For the first time, we could directly determine timing and rate of Chernozem/Phaeozem formation in Central Europe by dating bioturbation as the dominant soil forming process. Accordingly, Chernozem/Phaeozem formation started at the latest in the Early Holocene prior to Neolithic settlement indicating a natural origin of Central German Chernozems/Phaeozems, and Chernozem/Phaeozem formation ceased around 6–5 ka when the regional climate became more humid. Our effective soil reworking rates show that earthworm bioturbation in Chernozems/Phaeozems is more intense than ant-dominated bioturbation, but significantly less intense than bioturbation by lugworms or ploughing. The latter effect allows to identify prehistoric ploughing in paleosols.

## Introduction

Soils provide important ecosystem services such as biomass (food) production, water filtration or a high biodiversity (e.g.^1^), and archive our geological and archaeological heritage (e.g.^2,3^). Black-coloured humus-rich Chernozems and closely related Phaeozems^4^ globally cover 230 and 190 million hectares, respectively^5^. They contain ≥ 1.0% (chernic horizon of Chernozems) and ≥ 0.6% (mollic horizon of Phaeozems) organic carbon in their A horizon^4^, and are among the most fertile soils of the world^6,7^. Hence, they were often settled early on by farming societies in Central and Eastern Europe, and used for agriculture over several millennia^8–10^. Despite their particular importance for food supply and carbon sequestration, their origin and age remain controversial^11–14^.

**Figure 1:**
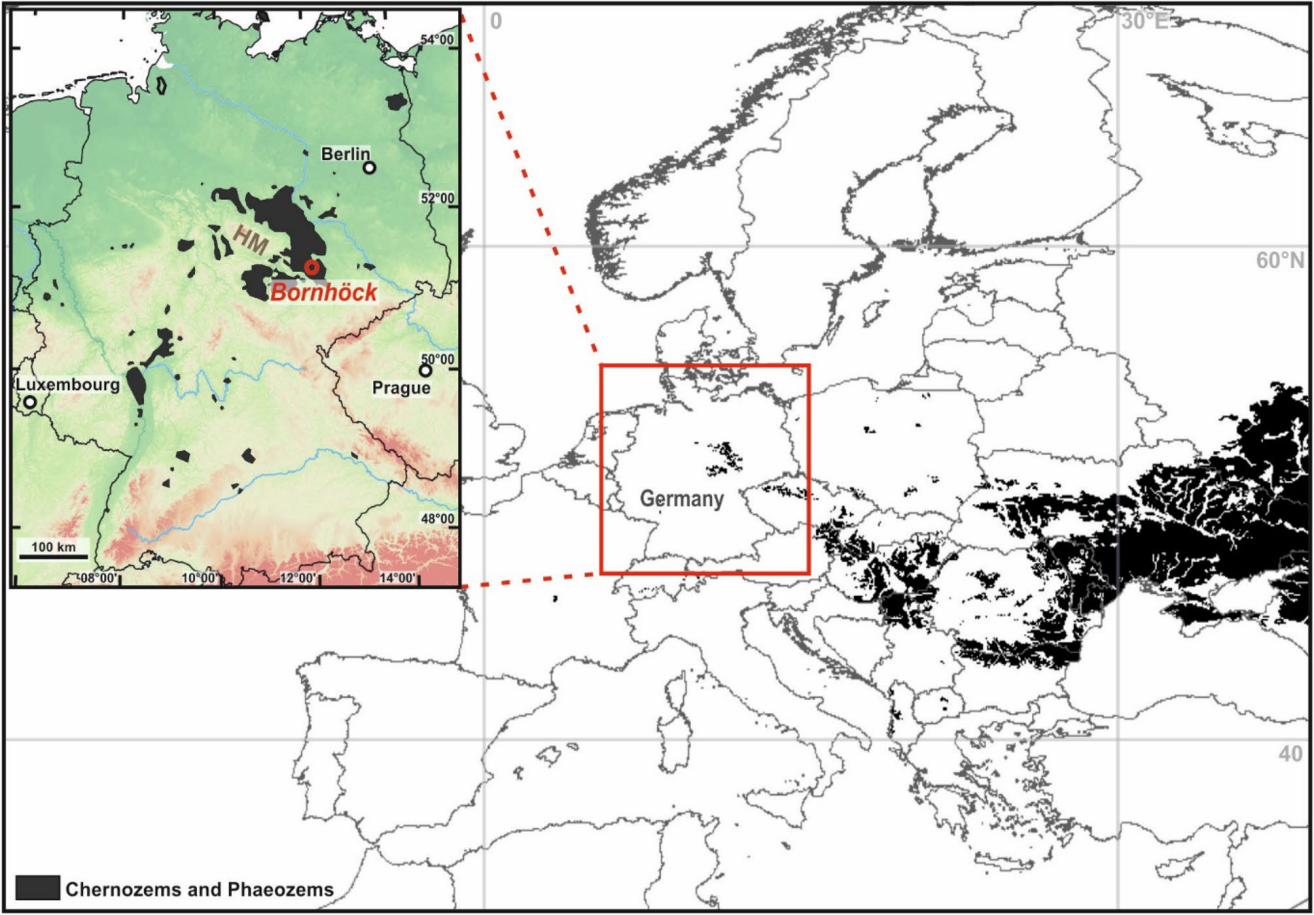
Distribution of Chernozems and Phaeozems in Europe (black colouring; taken from FAO/IIASA/ISRIC/ISSCAS/JRC 2012: Harmonized World Soil Database (v. 1.2). FAO Rome, Italy and IIASA, Laxenburg, Austria). Inset: The site Bornhöck in the Chernozem/Phaeozem area of Central Germany (red circle). The distribution of Chernozems/Phaeozems is shown in black, after Eckmeier et al.^12^ (modified). HM = Harz Mountains.

In Europe, the westernmost area of widespread Chernozems/Phaeozems is in Central Germany (Fig. 1). For most Chernozems/Phaeozems in other German regions an anthropogenic origin linked with fire use and/or input of combustion residues or marine biomass was demonstrated^15–17^. In contrast, most authors suggest a natural formation of the Central German Chernozems/Phaeozems prior to Neolithic settlement. This was likely linked with intense bioturbation as indicated by krotovinas and corresponding structures in micromorphological thin sections^18^, and probably favoured by calcareous Quaternary sediments such as loess and loess derivatives in combination with the relatively dry subcontinental climate in the lee of the Harz Mountains with annual precipitation between 440 and < 580 mm mm/a (^14,19^, Fig. 1). However, this hypothesis is still controversial, as Gehrt^13^ and Dreibrodt et al.^11^ suggest a general anthropogenic Chernozem/Phaeozem formation in Central Europe, which is either related to anthropogenic input of charred organic matter or to agriculturally fostered activity of anecic earthworms. One main reason for these ambiguities is that radiocarbon dating of these soils, the preferred geochronological method so far, only dates apparent mean residence times of soil organic matter (e.g.^20^) and therefore provides only minimum ages for the onset of Chernozem/Phaeozem formation^12,21^. Therefore, the timing and rates of Chernozem/Phaeozem formation are still largely unexplored, mainly because suitable methods to directly date soil formation were lacking.

However, such chronological information is mandatory to link soil processes or process rates to the corresponding environmental conditions. Some rapid soil processes such as overland soil erosion can be instrumentally measured (e.g.^22^), but most other soil forming processes occur much more slowly and require geochronological methods to constrain process rates (e.g.^23,24^). Over the last decade luminescence dating was increasingly used in this context (reviewed in^25^). One major advantage of this method is that the analysis of single sand-sized quartz or feldspar grains provides the necessary analytical resolution to identify different single-grain age populations associated, for example, to different soil sub-processes (e.g.^26,27^). or soil formation phases (e.g.^28^). During the last years versatile single-grain luminescence dating protocols were developed (e.g.^26^), alongside with computational tools to translate single-grain luminescence data into meaningful soil process rates (e.g.^29–31^). Hence, luminescence dating can provide valuable new insights into the timing of soil formation and underlying processes (e.g.^23^).

Here we used single-grain luminescence dating to resolve chronology and process rates of Chernozem/Phaeozem development by studying a Chernozem in Central Germany that was buried by the Early Bronze Age burial mound Bornhöck ca. 3.8 ka ago^32,33^. This covering by archaeosediments preserved the studied Chernozem from subsequent soil forming processes and agricultural activity, leaving it in an undisturbed state, which is exceptional for this fertile and intensively farmed region. Our aims were to: (i) Test the applicability of feldspar-based single grain luminescence dating for buried Chernozems/Phaeozems, (ii) determine beginning and end of the regional Chernozem/Phaeozem formation, (iii) derive soil reworking rates in this edaphic environment, and place these data in the context of palaeoenvironmental changes and regional human activity.

## Study area and study site

The *study area* is located in the Leipzig Basin that is filled with Paleogene to Quaternary loose sediments^34,35^. During the Quaternary, this lowland was repeatedly covered by ice shields of the Elsterian and Saalian glaciations, followed by (sand) loess deposition and periglacial reworking during the latest Saalian and Weichselian periods^36^. The region shows a fully humid warm temperate Cfb-climate with maximal summer precipitation^37^. Mean annual precipitation is around 551 mm, and average annual temperature around 9.1 °C (http://www.dwd.de). The study area lies in the south-eastern part of the Central German Chernozem/Phaeozem region, which had developed in the lee of the Harz Mountains (Fig. 1 inset)^38,39^. Within this region Chernozems and Phaeozems are spatially closely interfingered with each other^12^, so that both closely related soil types should have been formed by similar soil forming processes. The early Holocene climate was relatively warmer and drier and more subcontinental compared with today, followed by more humid conditions since ca. 6.7 ka and especially since 5.8 ka^40^. Agricultural land use started ca. 7.5 ka during the early Neolithic, and subsequently the region was nearly continuously inhabited with varying intensities^41^.

The *study site* is located on a flat Saalian moraine plateau (51°24′47.82″N, 12°5′4.61″E; 110 m a.s.l.). The Early Bronze Age burial mound Bornhöck was erected ca. 3.8 ka ago with a diameter of ca. 65 m and a height of 12–13 m, and grew in the Middle Ages and early modern times to almost 90 m in diameter and 15 m height^33^. During the second half of the nineteenth century it was completely destroyed^32^. However, the buried Chernozem soil remained protected against subsequent agricultural activity by a cover of archaeosediments of several decimetres until today. Bioturbation by macro- and megafauna hardly occurred after burial, as evidenced by the distinct layer boundary between the Bronze Age surface and the covering archaeosediments. In this area, Chernozems/Phaeozems had developed mainly in a 50 to 30 cm thick sand loess layer probably originating from the latest Pleistocene that is separated by a stone layer from the underlying Saalian glacial moraine deposited > 130 ka ago^36,42^.

## Methods

### Fieldwork

**Figure 2:**
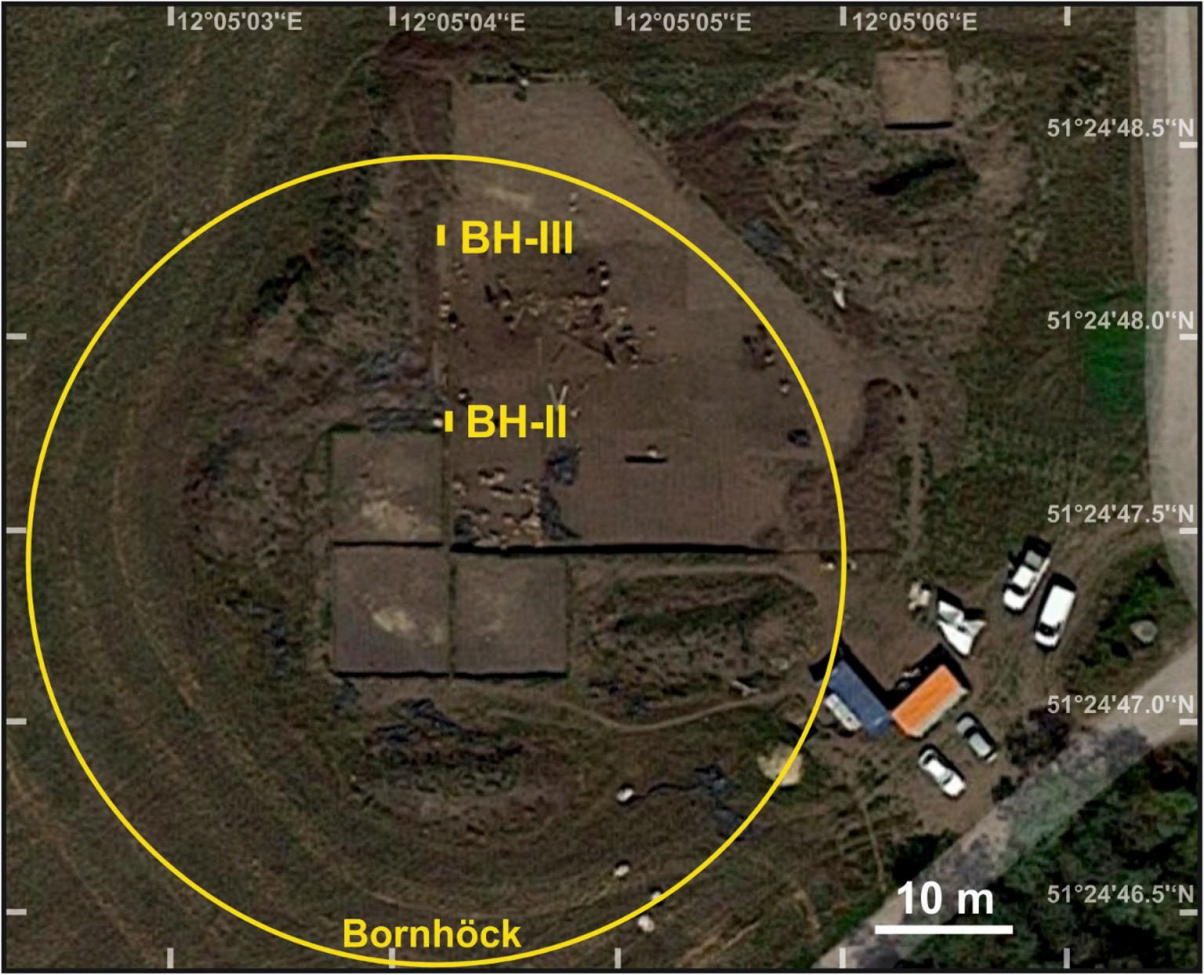
Aerial view of the Bornhöck area with positions of the two sampling sites (Google Earth 08.10.2015).

Two buried Chernozem soil profiles (BH II and III) were studied during ongoing excavations of the State Office for the Preservation of Archaeology Saxony-Anhalt in July 2018. Profile BH II was located in the northern centre of the former burial mound, and profile BH III near its northern margin in a distance of ca. 24 m from profile BH II (Fig. 2). The profiles were described according to Ad Hoc AG Boden^43^.

### Luminescence dating

All luminescence analyses were performed at the Netherlands Centre for Luminescence dating (NCL) at the Wageningen University and Research Centre.

#### Sampling and sample preparation

**Figure 3:**
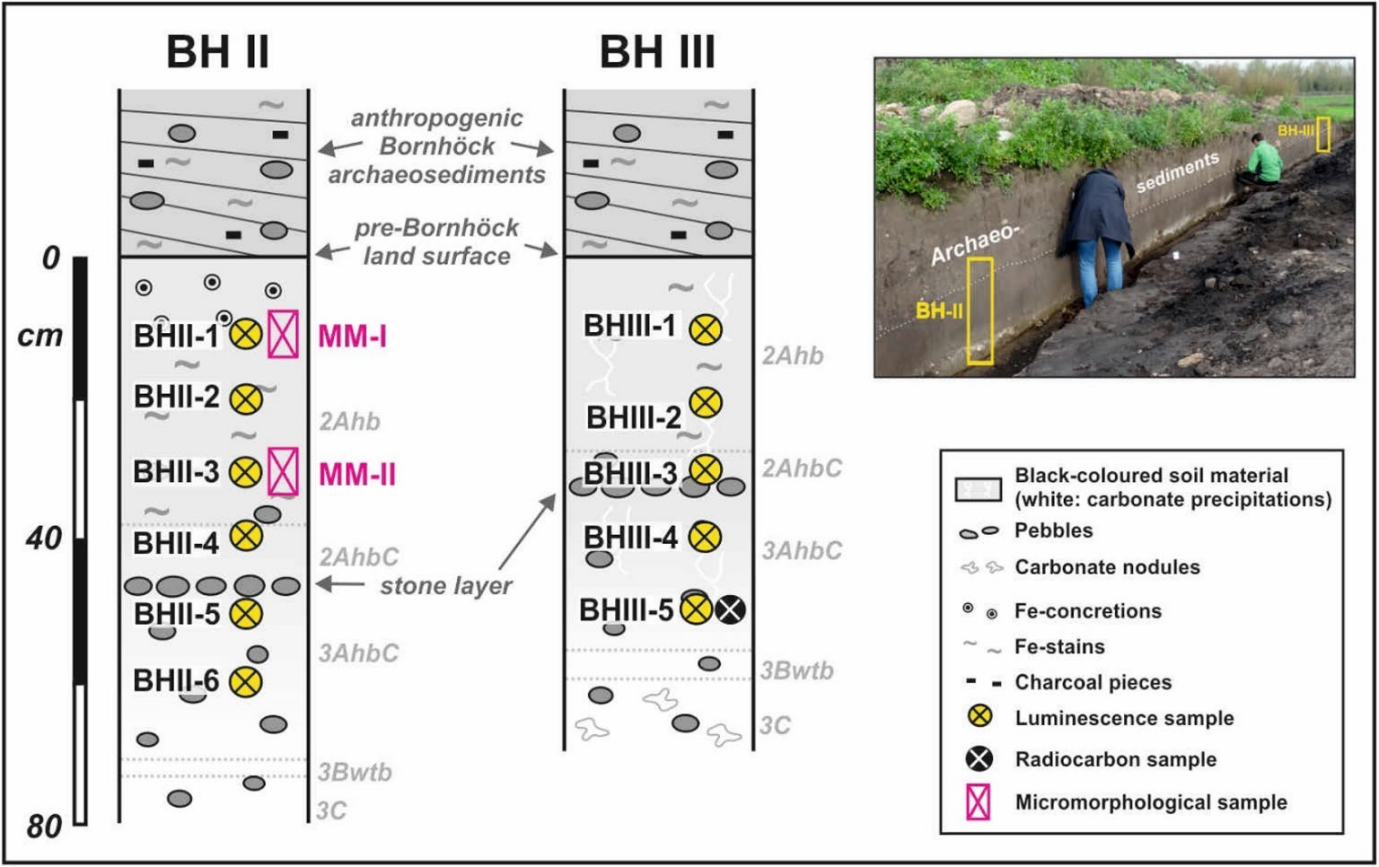
Soil profiles BH II and BH III with positions of the luminescence and micromorphology samples. Inset: Photo of the two studied profiles seen from southeast.

Six samples from profile BH II and five samples from profile BH III were taken with distances of about 10 cm. Samples BHII-1–BHII-4 and BHIII-1–BHIII-3 were taken from the upper sand loess layer above the stone layer, and samples BHII-5–BHII-6 and BHIII-4–BHIII-5 from the underlying Saalian moraine (positions see Fig. 3). The samples for equivalence dose determination were taken from freshly cleaned outcrop walls during night and directly packed into light-proof plastic bags. Sample preparation was performed under subdued orange light: the sand fraction 212–250 μm was obtained by sieving, treated with 10% HCl for 60 min to remove carbonate and subsequently with 10% H_2_O_2_ solution overnight to remove organic matter (OM). Using LST (lithium heteropolytungstate) heavy liquid of 2.58 g/cm^3^ the K-rich feldspar fraction (lighter) and quartz-rich fraction (heavier) were separated from each other, and the latter was etched with 10 and 40% HF for 45 min to remove the outer α-irradiated rim of the quartz grains and remaining feldspars.

During daylight, extra sample material was taken for dose rate determination including the estimation of OM and water content (WC). These samples were weighed, ashed at 400 °C and weighed again to determine the OM content. Subsequently, prior to the dose rate measurements these samples were milled and mixed with molten wax.

#### Equivalence dose determination

For the equivalent dose (D_e_) measurements, individual feldspar and quartz grains were loaded onto aluminium single-grain discs with a 10 × 10 grid of 300 μm holes. All luminescence measurements were performed using an automated Risø TL/OSL reader (DA15) equipped with a dual-laser single-grain attachment and a calibrated ^90^Sr/^90^Y beta source (dose rate 0.10 ± 0.004 Gy/s).

Single-grains from the K-rich feldspar fraction were stimulated for 2.0 s with a 150 mW 830 nm IR laser, and their emission around 410 nm was detected through a LOT/ORIEL D410 interference filter. The measurements were performed using the single-grain pIRIR protocol of Reimann et al.^26^ with some modifications of the preheat and pIRIR stimulation temperature. A preheat was applied for 60 s at 175 °C, and single-grain IRSL stimulation was carried out at 50 °C (IRSL_50_), and a subsequent pIRIR stimulation at 150 °C (pIRIR_150_). The regenerative dose response curves were constructed by irradiating the grains with 7.6, 15.2, 30.3, 60.6 and 121.2 Gy, and using an exponential fit through the origin with 100 Monte Carlo repeats for error calculations. The laboratory irradiation was limited to a maximum of 121.2 Gy to save machine time, since this study focussed on Latest Weichselian to Holocene soil mixing processes. Single-grains from the quartz-rich fraction were stimulated for 1.0 s with a 10 mW 532 nm laser, and the signals were detected through a 7.5 mm Hoya U-340 detection filter. The measurements were performed using a single-grain protocol comparable to Reimann et al.^44,45^, and the measurement steps included a 10 s preheat at 240 °C, a cut-heat at 220 °C and a single grain OSL stimulation at 125 °C. Similar to the feldspars, the regenerative dose response curves were constructed by irradiating the grains for 7.6, 15.2, 30.3, 60.6 and 121.2 Gy, and using an exponential fit through the origin with 100 Monte Carlo repeats for error calculations. All quartz and feldspar grains with a signal were accepted that fulfilled the following criteria: (i) a relative test dose error < 20%, (ii) a recycling ratio < 20% from unity, (iii) a maximum recuperation < 15% of the natural sensitivity, (iv) monotonically growing dose response curves. Prior to the measurements, single-grain dose recovery tests were performed on samples BHII-3 and BHIII-1, providing ratios of 0.99 ± 0.02 *(n* = *104)* and 1.00 ± 0.03 *(n* = *53)* for feldspar, and of 1.02 ± 0.05 *(n* = *17)* and 0.96 ± 0.04 *(n* = *19)* for quartz, respectively. These results confirmed the general suitability of the analysed K-rich feldspar and quartz fractions for single-grain luminescence analyses.

#### Handling of the equivalent dose data

To benchmark the performance of the feldspar single-grain pIRIR data, the Central Age Model (CAM^46^) was applied to the feldspar pIRIR_150_ and parallel quartz OSL single-grain D_e_ distributions of samples BHII-3 and BHIII-1, and the dose rate calculation outlined below was used to calculate the corresponding CAM ages. The feldspar single-grain pIRIR protocol is typically more measurement time efficient than quartz OSL single-grain measurements, as typically more than 10 times more K-rich feldspar grains provide a sufficient luminescence signal compared to quartz grains^26^.

To calculate the palaeodoses of the samples associated to the end of intensive light-exposure by intensive soil mixing, i.e. the so-called stabilisation age^47^, the bootstrapped Minimum Age Model (bootstrapped MAM^48^), was applied to the feldspar pIRIR_150_ single-grain D_e_ distributions (e.g.^49,50^). The input parameter sigma_b was set to 31 ± 5% following the recommendations of Brill et al.^50^.

#### Dose rate determination

The activity concentrations of the U and Th decay chains as well as the activity of ^40^K were measured with high-resolution gamma spectrometry. The β and γ dose rates were calculated using the conversion factors of Guérin et al.^51^. Due to strong drying of the samples in the archaeological trench that had been open for several months, the water contents (WCs) were estimated to 20.0 ± 7.5% per mass for the upper samples and to 22.0 ± 7.5% per mass for the samples deeper in the soil profiles. These estimates are based on well-established soil physical parameters, namely the field capacity and welding point for loessic soils with predominantly silty texture as upper and lower WC scenarios, respectively. Based on Smedley et al.^52^ the internal K-content of the feldspar single-grains was assumed to 10 ± 2% and the internal Rb-content to 400 ± 100 ppm. A dose rate contribution of 0.050 ± 0.025 Gy ka^−1^ associated to alpha irradiation of the outer part of the grain was also incorporated.

The cosmic dose rates were calculated according to the equation of Prescott and Hutton^53^. To obtain a meaningful estimate for the effective thickness of the overburden through burial time we had to best-estimate the effective burial depth in an iterative manner, since the burial depth of the samples before and after construction of the Bornhöck burial mound around 3.8 ka was significantly different^32^. The input for the iterative approach was derived from the geoarchaeological information provided in "Study area and study site" section (above). This iterative approach included the following assumptions: (i) the burial depth for the post-Bornhöck period was assumed to 13 m and 6.5 m for the BH-II and BH-III samples, respectively, (ii) instant and not gradual burial by the burial mound was assumed, (iii) the construction time of the burial mound was estimated to 3.8 ka, (iv) for the pre-Bornhöck time the surface of the buried palaeosol served as a reference to calculate the burial depth, (v) the period after removal of the burial mound (last ca. 150 years) was not considered. Starting with these assumptions we first calculated the apparent burial ages only considering the post-burial depths. The resulting apparent burial ages were used to make a first estimate regarding the relative time contributions of the pre- and post-burial depths to the effective burial depth, and this first estimate was used to refine the burial ages. This was iterated until burial ages and burial depths remained unchanged. The final effective burial depths are reported in Table 2.

### Radiocarbon dating

One soil sediment sample was taken for radiocarbon dating from a depth of 50 cm from profile BH III, i.e. from the lowest part of the Ahb horizon of the buried Chernozem to obtain the oldest possible age of OM of that soil (position see Fig. 3). The sample was prepared with the Acid-Base-Acid method^54^, and the bulk OM after removing the soluble humic acids was dated in the CEZ radiocarbon laboratory Mannheim. The age was calibrated using the software SwissCal and the Intcal20 calibration curve^55^.

### Micromorphology

For micromorphological investigation, two oriented undisturbed soil samples (MM-I: upper sample, MM-II: lower sample; see Fig. 3) were taken from the Ahb horizon of profile BH II. After air-drying and impregnating with Oldopal P 80-21, hardened blocks were cut and sliced into 90 * 60 mm thin sections and scanned with a flatbed scanner. The thin sections were described at 50–400 magnification under a polarizing light microscope mainly using the terminology of Stoops^56^.

## Results

### Fieldwork (Fig. *3*)

#### Profile BH II

The buried Chernozem is covered by about 80 cm of layered black-coloured archaeosediments of the former burial mound, containing many pebbles, charcoal and partly banded Fe-concretions. A 2Ahb horizon represents the upper 38 cm of the buried Chernozem. Fe-concretions up to 4 mm are found in its uppermost 10 cm and below Fe-precipitations occur along root channels, probably reflecting the stagnation of water on the border between the loose overlying archaeosediments and the denser paleosol. The black colour of 10YR2/1 changes at 28 cm to very dark brown (10YR2/2), with single brownish Fe-stains and occasional pebbles. These dark colours qualify this horizon as “chernic” and hence this paleosol as a Chernozem^4^. Between 38 and 70 cm the colour changes from very dark brown (10YR2/2) in the upper to dark yellowish brown (10YR4/4) in the lower part (2AhbC/3AhbC horizon). Here, small black clay coatings are found in macropores. A stone layer at ca. 45 cm separates the sand loess from the underlying Saalian moraine. Consequently, above the stone layer there are very few pebbles and the material consists of clay loam, whereas below this layer the amount of pebbles increases and the texture changes to sandy clay loam^57^. A dark yellowish brown (10YR 4/4) 3Btwb horizon follows between 70 and 73 cm, and a yellowish brown (10YR 5/4) 3C horizon between 73 and 80 cm. While the Ck horizon is strongly calcareous representing the original carbonate content of the Saalian moraine, all material above 73 cm is non-calcareous.

#### Profile BH III

The buried Chernozem is covered by about 20 cm decimetres of layered and pebble-rich black-coloured archaeosediments from the former burial mound. The 2Ahb horizon (thickness 27 cm) has some brownish Fe-precipitations with downward increasing quantity, what similar with profile II reflects the stagnation of water on the border between the loose overlying archaeosediments and the denser paleosol. This horizon is black (10YR 2/1) down to 20 cm, and very dark brown (10YR2/2) within the lower 27 cm, what in agreement with profile BH II qualifies this horizon as “chernic”. The colour of the 2AhbC/3AhbC horizon (27–56 cm) changes from very dark grey (5YR3/1) in the upper part to brown (7.5YR4/4) in the lower part. A stone layer at 32 cm within this horizon separates the overlying sandy loess from the underlying Saalian moraine. Above this layer, pebbles are occasionally found and the material consists of clay loam, whereas below the pebble amount increases and the texture changes to sandy clay loam^57^. Below the 2AhbC/3AhbC horizon a brown (7.5YR4/4) 3Btwb horizon follows between 56 and 60 cm, and between 60 and 70 cm a yellowish brown (10YR5/4) 3C horizon. The latter is strongly calcareous representing the original carbonate content of the Saalian moraine, and single carbonate nodules must have formed by leached carbonate from the overlying sediments. In contrast, all overlying Chernozem horizons are weakly calcareous. The buried Chernozem was slightly carbonatic only in the border area of the former burial mound. Hence, this carbonate content was probably caused by secondary carbonatization due to leaching of the white lime paint of the former burial mound^32^. Finally, the covering archaeosediments are non-calcareous, probably due to sub-recent agricultural activity.

### Luminescence dating

#### Performance of the single-grain quartz and feldspar approaches

The exemplary comparison of the CAM-D_e_ based on single-grain quartz OSL with that based on single-grain feldspar pIRIR of samples BHII-3 and BHIII-1 confirms a higher percentage of suitable grains for feldspar single-grain luminescence analyses (Table 1): In agreement with previous studies from the North European Plain (e.g.^44,45,58^) only ~ 2.6% of the single-grain quartz grains provided a suitable OSL signal, i.e. for most quartz grains too low OSL signal intensity was the limiting factor. In contrast, ~ 50% of the feldspar single grains provided a sufficient pIRIR signal, confirming previous studies (e.g.^26,45^). Therefore, our data indicate that the single-grain feldspar pIRIR approach is ~ 20 times more efficient in measuring D_e_ data than quartz OSL, being a major advantage when studying settings with potentially complex D_e_ distributions such as archaeological sites or soils.

**Table 1:** Exemplary comparison of quartz OSL single-grain ages versus feldspar pIRIR single-grain ages.

Sample field ID	NCL ID	Single-grain approach	Depth [cm]	n (measured/accepted)^a^	CAM D_e_ [Gy]^b^	Total dose rate [Gy/ka]	Age [ka]
BHII-3	NCL-1119003	Quartz OSL	30	1199/31	21.6 ± 2.9	2.50 ± 0.14	8.65 ± 1.25
		Feldspar pIRIR	30	271/143	26.4 ± 1.0	3.29 ± 0.20	8.02 ± 0.57
BHIII-1	NCL-1119007	Quartz OSL	10	1213/31	17.0 ± 1.2	2.08 ± 0.12	7.36 ± 0.92
		Feldspar pIRIR	10	283/131	19.3 ± 0.8	2.87 ± 0.19	6.72 ± 0.52

^a^Grains in the single-grain discs were counted under the microscope. Accepted grains are grains that met the acceptance criteria outlined in main text.

^b^The Central Age Model (CAM^46^) was used for calculation.

The weighted mean CAM D_e_ were used for comparison (Table 1): The quartz OSL and feldspar pIRIR CAM burial ages of both samples agree with each other within the 1-sigma confidence interval. This suggests that soil mixing during Chernozem/Phaeozem formation sufficiently resets the pIRIR luminescence signal. Furthermore, anomalous fading which would cause significant age underestimation of the pIRIR feldspar ages is apparently sufficiently dealt with by the pIRIR measurement protocol^59^. However, both mid-values of the pIRIR feldspar ages are slightly younger than the corresponding quartz OSL ages, possibly hinting to a very small amount of anomalous fading. To further test this we performed additional laboratory fading tests according to Auclair et al.^60^. The results, however, are inconclusive as no clear trend of signal loss with storage time was observed. Therefore, we decided to not apply a fading correction to our single-grain feldspar pIRIR ages.

#### *Single-grain feldspar pIRIR D*_*e*_* distributions*

**Figure 4:**
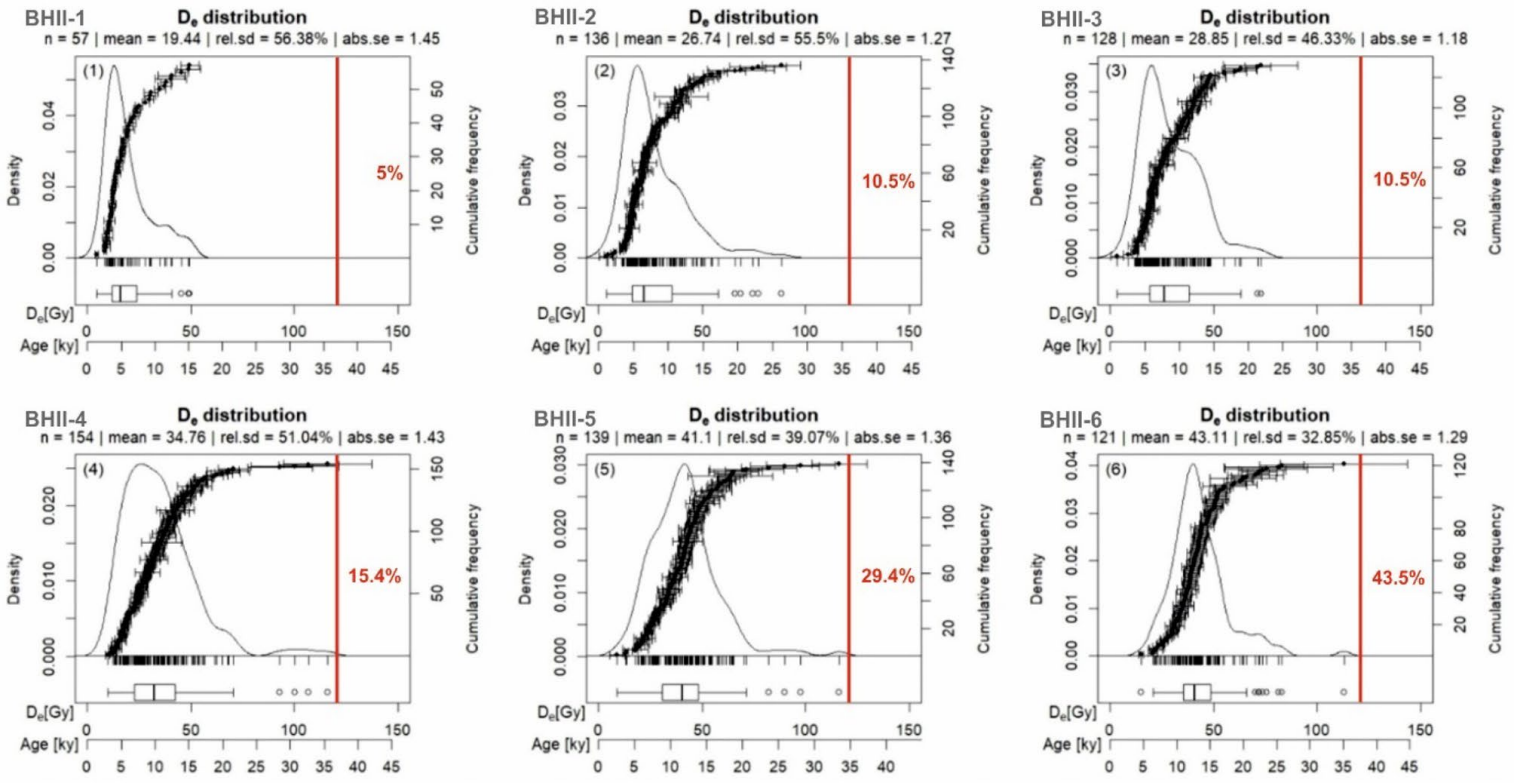
KDE plots of the single-grain feldspar pIRIR D_e_ distributions for the six samples from profile BH II. For better comparison of the samples we also converted the conventional D_e_ x-axis to an apparent age axis using the sample dose rates. The red vertical lines indicate 121.2 Gy, which was the maximum irradiation point (see “Methods” section for details). The percentage of grains outside this 121.2 Gy threshold is also indicated in red.

**Figure 5:**
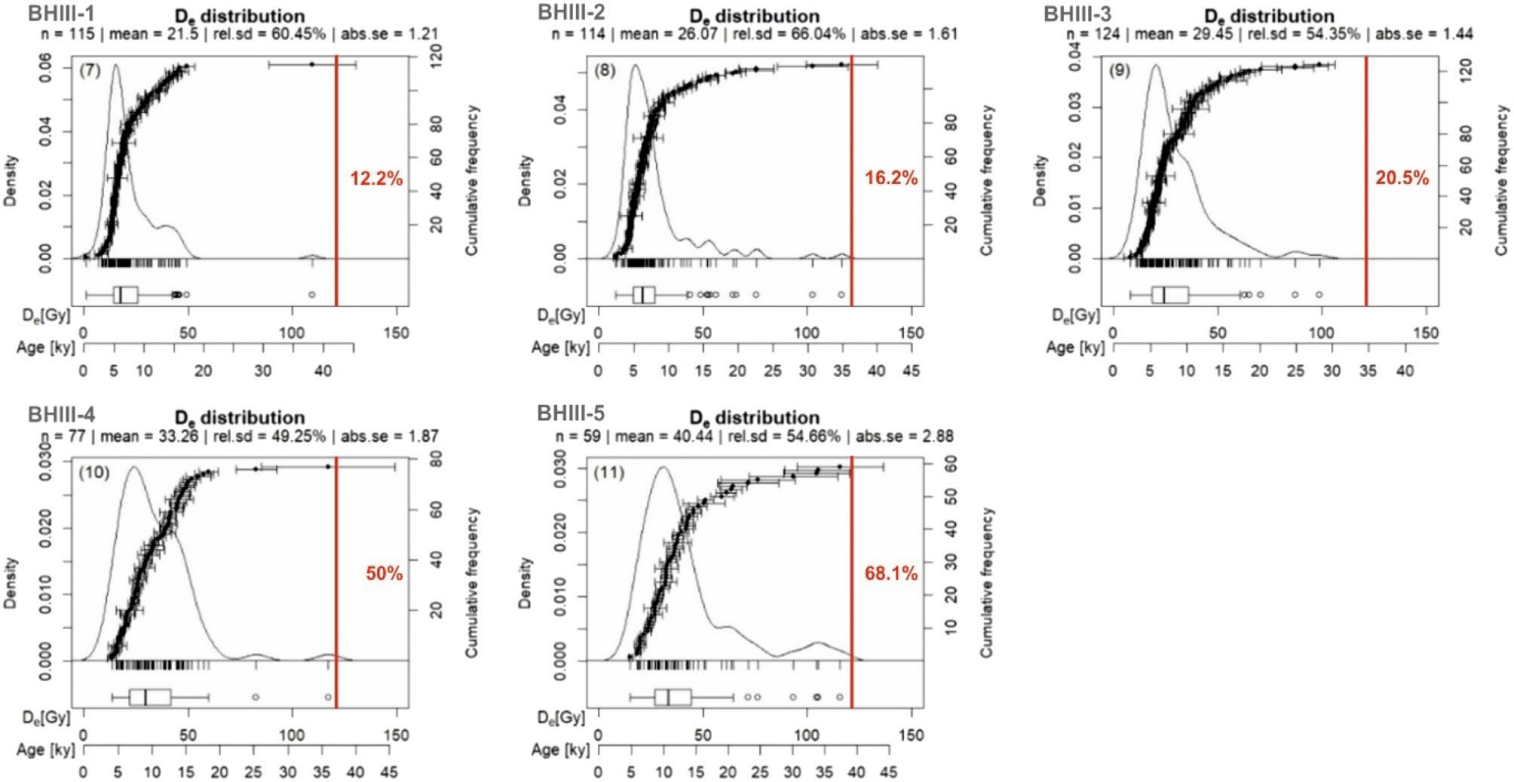
KDE plots of the single-grain feldspar pIRIR D_e_ distributions for the five samples from profile BH III. For better comparison of the samples we also converted the conventional D_e_ x-axis to an apparent age axis using the sample dose rates. The red vertical line indicates 121.2 Gy, which was the maximum irradiation point (see method section for details). The percentage of grains outside this 121.2 Gy threshold is also indicated in red.

The single-grain feldspar pIRIR D_e_ distributions for both profiles are relatively broad (Figs. 4, 5), which is typical for D_e_ distributions containing both originally deposited and post-depositional grains associated to soil formation (e.g.^61^). Furthermore, mean and median of the D_e_ distributions and the proportion of “old” grains above 121.2 Gy systematically increase with depth for both profiles (Figs. 4, 5). These observations presumably suggest that the impact of post-depositional processes, surfacing grains, diminishes with depth.

#### Timing of intensive soil reworking

**Figure 6:**
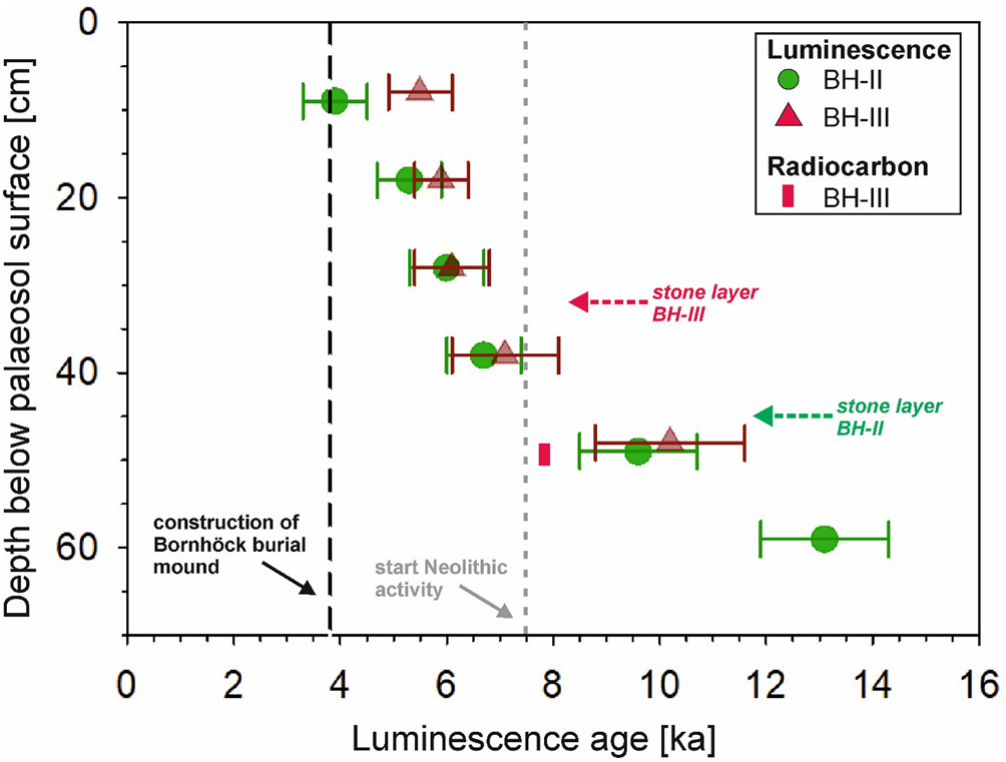
Age-depth plots for both soil profiles BH II and BH III. The luminescence stabilisation ages were calculated based on the single-grain feldspar pIRIR palaeodoses associated with sample stabilization ages.

Establishing meaningful palaeodoses and ages from the broad single-grain feldspar pIRIR D_e_ distributions (Figs. 4, 5) is challenging. Van der Meij et al.^47^ suggested that applying the bootstrapped MAM of Cunningham and Wallinga^48^ to broad and/or complex D_e_ distributions allows to reliably extract palaeodoses associated with sample stabilization, i.e. to obtain the timings when sample rejuvenation by recently surfaced grains significantly dropped. For soil samples this stabilization age is associated with the end of intensive soil mixing e.g. by bioturbation or ploughing. The stabilization ages of our samples are listed in Table 2, and shown in Fig. 6.

**Table 2:** Single-grain pIRIR stabilisation ages, and the parameters that were applied for age calculation.

Field ID	Lab. ID	Depth below paleosol surface [m]	Effective burial depth [m]^a^	U [Bq/kg]^b^	Th [Bq/kg]^b^	K [Bq/kg]^b^	Palaeodose [Gy]^c^	Dose rate [Gy/ka]	Age [ka]	Systematic/random error
BHII-1	NCL-1119001	0.09	12.7	31.7 ± 0.4	34.8 ± 0.9	601.0 ± 11.9	12.5 ± 1.7	3.20 ± 0.20	3.9 ± 0.6	0.27/0.54
BHII-2	NCL-1119002	0.18	9.4	31.0 ± 0.4	35.0 ± 0.9	600.1 ± 11.9	16.9 ± 1.7	3.24 ± 0.20	5.3 ± 0.6	0.35/0.53
BHII-3	NCL-1119003	0.28	8.3	31.0 ± 0.4	34.3 ± 0.9	588.7 ± 11.7	18.9 ± 1.8	3.14 ± 0.20	6.1 ± 0.7	0.40/0.58
BHII-4	NCL-1119004	0.38	7.5	29.3 ± 0.4	31.7 ± 0.9	575.1 ± 11.4	20.5 ± 1.8	3.08 ± 0.20	6.7 ± 0.7	0.45/0.59
BHII-5	NCL-1119005	0.49	5.4	34.8 ± 0.4	36.1 ± 1.0	597.5 ± 11.8	31.7 ± 3.0	3.30 ± 0.21	9.7 ± 1.1	0.63/0.92
BHII-6	NCL-1119006	0.59	4.2	27.0 ± 0.4	31.1 ± 0.9	561.3 ± 11.2	40.6 ± 2.3	3.11 ± 0.20	13.2 ± 1.2	0.88/0.76
BHIII-1	NCL-1119007	0.08	4.5	22.8 ± 0.3	26.4 ± 0.8	525.0 ± 10.5	15.7 ± 1.2	2.86 ± 0.19	5.5 ± 0.6	0.31/4.52
BHIII-2	NCL-1119008	0.18	4.3	33.4 ± 0.4	35.5 ± 0.9	604.3 ± 11.9	19.2 ± 1.0	3.27 ± 0.20	5.9 ± 0.5	0.36/4.25
BHIII-3	NCL-1119009	0.28	4.2	31.8 ± 0.4	33.9 ± 0.9	655.6 ± 12.8	20.3 ± 2.1	3.35 ± 0.21	6.1 ± 0.7	0.38/4.15
BHIII-4	NCL-11190010	0.38	3.7	30.9 ± 0.4	29.7 ± 0.7	584.3 ± 11.5	22.2 ± 2.7	3.11 ± 0.20	7.2 ± 1.0	0.46/3.66
BHIII-5	NCL-11190011	0.48	2.7	28.2 ± 0.4	28.6 ± 0.8	533.9 ± 10.7	29.9 ± 3.6	2.97 ± 0.19	10.2 ± 1.4	0.62/2.74

^a^The effective burial depth was iterated based on the sample position with respect to the hypothetical surface of the Bornhöck burial mound and the surface of the pre-Bornhöck palaeosol. For details, see main text.

^b^Activity concentrations were measured using high resolution gamma spectrometry. See main text for details.

^c^The palaeodose is based on the bootMAM^48^ applied to the corresponding pIRIR_150_ single-grain D_e_ distributions. See main text for details.

Both age-depth plots indicate stabilization of the samples below the stone layer between ~ 13.2 ka and ~ 7.2 ka during the late Glacial to Middle Holocene. In contrast, the samples above the stone layer show Middle to Late Holocene stabilization ages between ~ 7.2 and ~ 3.9 ka^62^.

#### Luminescence based soil reworking rates

From the age-depth data we calculated effective soil reworking rates according to Reimann et al.^26^. This approach calculates soil mixing based on age and depth of a luminescence sample^24,63^, also considering the percentage of grains that participated in the mixing process to obtain a more realistic measure of soil mixing. Reimann et al.^26^ showed a relatively simple case of soil formation in granitic bedrock, where the percentage of unmixed grains could be straightforwardly estimated by the amount of infinitely old bedrock grains showing saturation ages. In our setting soil formation predominantly occurred in latest Pleistocene sand loess^42^ that was not necessarily deposited much earlier than post-depositional bioturbation of the mineral grains. Therefore, to approximate the fraction of mixed and unmixed grains we characterised any grain inside the 2-sigma band of the MAM D_e_ population as mixed, and any grain outside (i.e. significantly older) as unmixed. Unlike previous attempts working on surface soils (e.g.^24,26^), we also had to include the age of the palaeosurface (3.8 ka) in our calculations.

**Figure 7:**
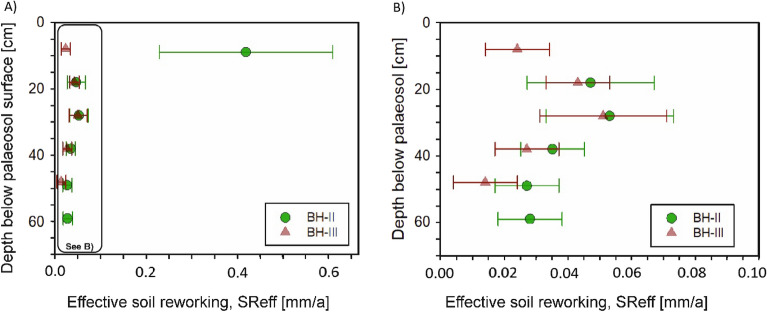
Effective soil reworking rates for the two soil profiles BH II and BH III. The inset of (**A**) is shown in (**B**). The values of the rates can be found in Table SOM-1.

All samples but sample BHII-1 show effective soil reworking rates between ~ 0.014 and ~ 0.053 mm/a (Fig. 7; Supporting Table 1). In contrast, the rate of sample BHII-1 is an order of magnitude greater at 0.42 ± 0.19 mm/a, which is likely related to anthropogenic mixing (ploughing) up to burial mound construction 3.8 ka ago. For the other samples, rates peak at 30 cm depth and appear to decrease below and above (especially for profile BH III).

### Radiocarbon dating

Radiocarbon dating of soil OM from the 3AhbC horizon in profile BH III predates the Early Neolithic (Table 3).

**Table 3:** Results of radiocarbon dating. The error of the calibrated age is included within the given age range, since the error of the calibrated range is included in the calibration process using the software Oxcal with the latest dataset IntCal20^55,64^.

Profile and sample depth	Sample number	Material	δ^13^C (‰)	C (%)	^14^C age (year BP)	Calibrated ^14^C age (cal. ka BC)
BH III, 50 cm	MAMS-46351	Soil OM (bulk material after alkali solution)	− 39.7	0.2	6985 ± 44	6.0–5.7

### Micromorphology

**Figure 8:**
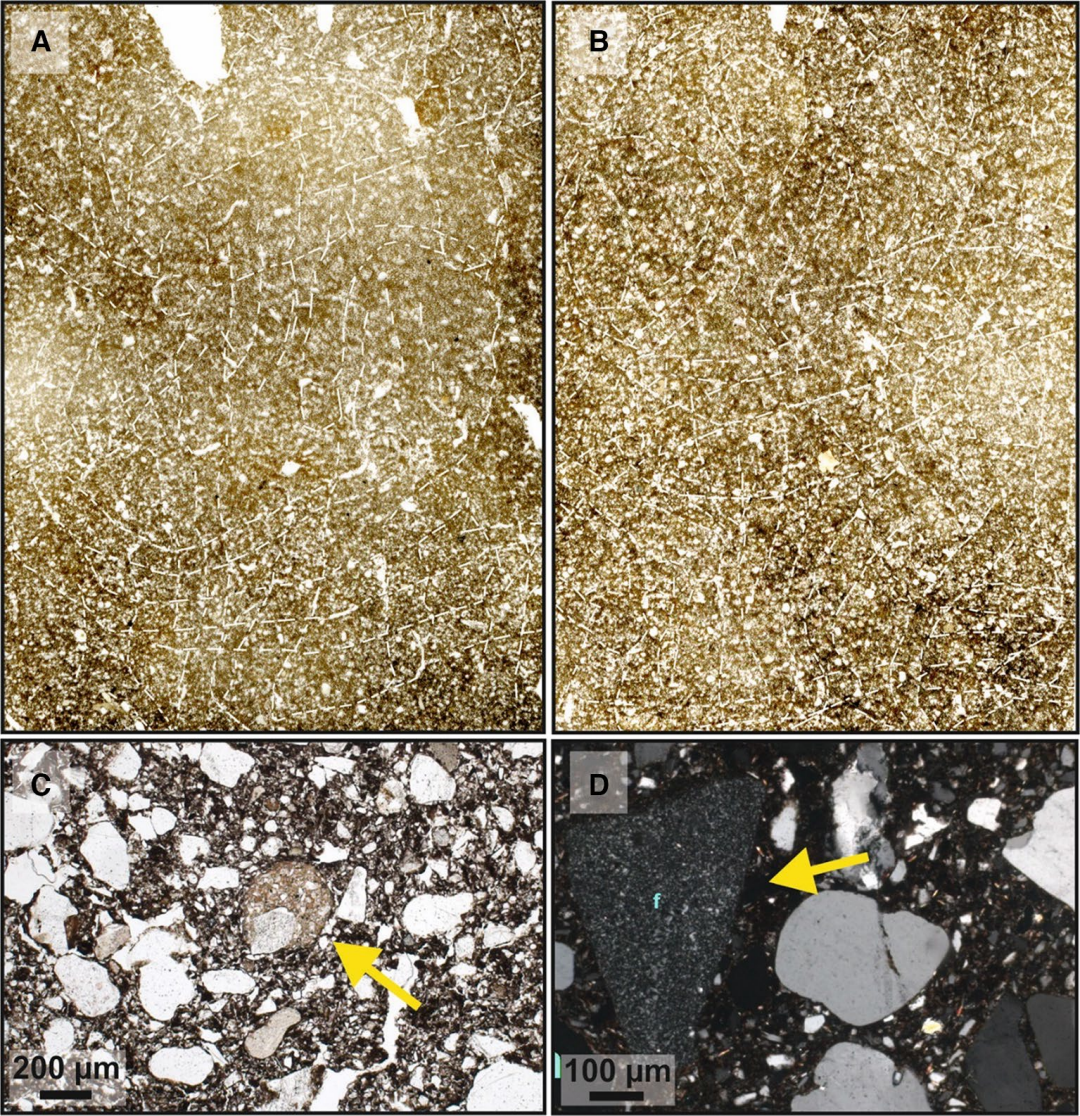
Results of the micromorphological analyses. (**A**) Micromorphological sample MM-I (size: 60*90 mm); (**B**) Micromorphological sample MM-II (size: 60 * 90 mm). Dashed lines in Figures A and B indicate the boundaries of passage features. Please also note the vertically, horizontally and diagonally oriented filled channels in both thin sections; (**C**) The yellow arrow marks a rounded fragment of clay-rich material (brownish areas) in sample MM-II. (plane polarized light); (**D**) The yellow arrow marks triangular flint in sample MM-II. Most other larger minerals are quartz (crossed polarized light).

Both thin sections MM-I and MM-II from profile BHBH II show numerous passage features, which were mainly produced by earthworms, snails and mollusks^65^. Their abundance indicates intense and nearly complete soil mixing by mesofauna (Fig. 8A, B). The passage features consist of discontinuously distributed and often compacted crumbs. The porosity is high and generally made up of (mamillated) vughs and channels. Partly accommodating planes in sample MM-I indicate a weakly developed subangular blocky microstructure. In lower sample MM-II numerous rounded aggregates up to 300 µm in diameter show a stipple speckled b-fabric and higher clay content than the surrounding soil mass (Fig. 8C). These also occur in much smaller quantities in upper sample MM I. Flint pieces (diameter up to 500 µm) occasionally occur in the coarse material of both thin sections, with a higher quantity in lower sample MM-II (Fig. 8D).

## Discussion

### Applicability of the feldspar-based luminescence protocol for dating buried Central German Chernozem/Phaeozem formation

An important prerequisite for reconstructing activity phases and rates of soil processes using luminescence is a vertical particle flux during soil formation that continuously produces bleached silicate (preferably sand-sized) grains (see review in^25^). Similar with former regional studies^12,19^, the micromorphological results clearly demonstrate intensive bioturbate mixing also for the studied buried Chernozem. Particularly, vertical movement during this process is demonstrated by a higher proportion of flints/cherts in the lower micromorphological sample MM-II compared to the upper sample MM-I, indicating that Saalian moraine material from below the stone layer must have been moved upwards into the upper sand loess layer during bioturbation. Also rounded clay-rich aggregates occurred especially in lower sample MM-II. We can exclude that these result from clay translocation that would have been easily detectable through clay coatings in Chernozem/Phaeozem horizons (e.g.^66^). However, as no fragments of clay coatings were found in either thin section, also these aggregates must have been transported upwards by bioturbation from the Eemian Bt horizon that had formed in the underlying Saalian moraine^36,42^. Altogether, our micromorphological analyses suggests a vertical particle flux caused by bioturbation, i.e. timing and rate of soil formation can be quantified by luminescence dating.

Our luminescence data clearly confirm that a single-grain resolution is required to study post-depositional soil mixing processes in detail (e.g.^24,26,61^), since even for the uppermost samples a notable number of grains yields ages that are linked with Pleistocene deposition phases (Figs. 4, 5). Hence, if measured as bulk, averaging effects would potentially obscure the genuine chronological information on either post-depositional mixing or the preceding deposition. The fraction of depositional, i.e. un-mixed grains increases with depth, indicating that bioturbation intensity is clearly depth-dependent, which agrees with previous observations in other settings^23^.

Comparing the quartz vs. feldspar single-grain results (Table 1 and associated paragraphs) confirms that feldspar single-grain measurements allow to quantify timing and rates of soil mixing processes^26^. Furthermore, the MAM pIRIR_150_ ages associated to the termination of intensive soil formation (see Fig. 6) fit very well with the age of burial mound construction ca. 3.8 ka ago. Our data confirm that for loose sediments with a low percentage of quartz luminescent grains, the measurement of feldspar single-grains using the pIRIR protocol^26^ is a versatile and more labour-efficient alternative.

Our analyses also support the previous study of van der Meij et al.^47^ that the termination of the most recent mixing activity phase—the so-called stabilisation age—can be reliably estimated with applying the bootMAM of Cunningham and Wallinga^48^ to single-grain D_e_ distributions. It seems that this statistical model could sufficiently capture the younger leading-edge populations of the corresponding single-grain pIRIR_150_ D_e_ distributions, since the resulting vertical successions of stabilisation ages in both profiles are internally consistent. Interestingly the stabilisation ages increase with depth for both profiles, which is not straightforward to interpret since bioturbation could affect grains in all depths during the whole soil formation period. Given that the likelihood of grains to be surfaced and thus bleached decreases with depth, the preservation potential for “older” surfaced grains should increase with depth. Hence, for deeper samples the larger proportion of older grains results in broader D_e_ distributions, and most likely also broadens the younger leading-edge D_e_ populations (see e.g. Figs. 4, 5).

We suggest that the upper part of the studied Chernozem was mainly controlled by a constant rejuvenation process of surfaced grains and thus provides a leading-edge D_e_ population dominantly reflecting the end of Chernozem soil formation, while the leading-edge D_e_ populations of the deeper soil samples reflect the older soil reworking history. In this context, considering luminescence age errors of roughly 10–20% (2-sigma confidence), the absence of a clearly separated pre-mixing population (Figs. 4, 5) suggests that the time gap between sand loess deposition and onset of soil formation could not have been larger than approx. 1–2 ka. Accordingly, the relatively mobile sand loess could only have been stabilized at earliest since the development of a certain vegetation cover at the start of the Bölling interstadial around 14.7 ka, and at latest at the end of the Younger Dryas around 11.7 ka prior to the establishment of a dense vegetation cover^42,67^.

### Start and end of Chernozem/Phaeozem formation in Central Germany

So far, timing and causes of Central European Chernozem/Phaeozem formation are still debated. Radiocarbon dating only indicates the mean residence times of OM rather than the start of Chernozem/Phaeozem formation. Therefore, the ages for Chernozem/Phaeozem-derived humic acids, bulk OM and burnt organic material widely vary between about 7.5 ka and some hundred years^15,68,69^, mostly postdating first Neolithic settlement since ca. 7.5 ka^70^. Furthermore, black-coloured soil formation in the context of Neolithic to Medieval human activity was demonstrated for several regions^15–17,71^. Gehrt^13^ suggests a generally anthropogenic origin of such soils in Central Europe. However, during archaeological excavations in Germany fully developed Chernozems/Phaeozems were observed below Early Neolithic settlements^72,73^. Accordingly, cryoturbated black-coloured soil horizons in Lower Saxony were interpreted as Late Pleistocene initial Chernozems^74^. Likewise, also for central Hesse Kühn et al.^66^ suggested Late Pleistocene initial Chernozem formation prior to the Laacher See volcanic eruption ca. 13 ka^75^. Regarding the termination of Chernozem formation, Meixner^10^ observed a degraded Chernozem below a colluvial deposit that, according to archeological finds, should have formed during or shortly after Early Neolithic settlement. In contrast, based on luminescence dating Kühn et al.^66^ suggested continuing Chernozem/Phaeozem formation in central Hesse until at latest 4.3 ± 0.3 ka. A weakly degraded Chernozem (signs of clay illuviation) below a Late Neolithic mound in Central Germany^76^ indicates a similar timing of the termination of Chernozem/Phaeozem formation before the Late Neolithic (4.8–4.2 ka^77^).

The single-grain luminescence age of 13.2 ± 1.2 ka for sample BHII-6 (Fig. 6), taken from the Saale moraine below the stone layer, possibly indicates the onset of bioturbation and thus Chernozem formation already during the latest Pleistocene. Alternatively, this age could also correspond to a grain population that was surfaced and bleached by cryoturbation processes during the Younger Dryas (12.9–11.7 ka^67^). In contrast, the Early Holocene ages of samples BHII-5 (9.7 ± 1.1 ka) and BHIII-5 (10.2 ± 1.4 ka), both taken from 50 cm depth, can only be linked to grain surfacing related to bioturbation. The slightly younger but consistent radiocarbon age of 8.0–7.8 cal. ka BP derived from bulk OM from the same depth indicates the link of bioturbation with organic matter accumulation, being a typical feature of Chernozem/Phaeozem formation (Fig. 6). As mentioned above, radiocarbon dating yields the mean residence time of OM^12^ and thus provides minimum ages of soil formation. Consequently, together with the presented luminescence ages this age thus shows that the Chernozem formation at our study sites must at latest have started in the Early Holocene ca. 11–9 ka ago, i.e. well before the Neolithic, which began around 7.5 ka^70^. Therefore, also supported by a partial spatial mismatch of Early Neolithic sites and the distribution of Chernozems/Phaeozems in Central Germany, our luminescence ages argue for a natural formation of these soils in Central Germany associated with intensive bioturbation^14,19^. Additionally they falsify the hypothesis of Gehrt^13^ that Chernozem/Phaeozem formation in Central Europe was generally linked with anthropogenic input of charred OM. Chernozem/Phaeozem formation was facilitated by the relatively dry and warm regional climate during the Early Holocene^40^ in combination with calcareous loose Quaternary sediments^19^. With regard to the finding that Chernozem/Phaeozem formation was linked to bioturbation caused by anecic earthworms^11^, our pre-Neolithic ages for the start of Chernozem/Phaeozem formation suggest that anecic earthworms must have found appropriate living conditions in this region already prior to human agriculture. Most of the stabilisation ages in the upper parts of the profiles (10, 20, 30 cm depth) are between ca. 5.3 and 6.1 ka (Fig. 6), indicating that intense regional Chernozem/Phaeozem formation must have ceased since then. These ages correspond to the end of Chernozem formation below the Late Neolithic mound in Werben, about 26 km to the south: The Chernozem was already slightly degraded during Late Neolithic burial ca. 1 ka later between 4.8 and 4.2 ka^76,77^. This is indicated by notable traces of clay illuviation not observed at our site (see “Micromorphology” section), although the Chernozem was burial several hundred years later compared to Werben^76^. According to Rogaar et al.^78^ a mean annual precipitation of ≥ 600 mm enhances clay illuviation. Hence, the clay coatings in the Ah horizon of Werben suggest higher precipitation during the mid-Holocene compared to the Bornhöck site which matches the recent mean annual precipitation pattern (Werben = 604 mm/a, Bornhöck = 551 mm/a; http://www.dwd.de).

In summary, these results suggest that natural Chernozem/Phaeozem formation in Central Germany lasted until the late mid-Holocene between about 6 and 5 ka. A similar timing for natural Chernozem/Phaeozem formation was found in central Hesse^66^, and may also apply to other loess-covered basins and leeward areas in Central Europe. Paleoenvironmental proxies from lake sediments of Salziger See suggest more humid conditions in Central Germany from ca. 6.7 ka and increasingly from 5.8 ka onwards^40^. Furthermore, well-resolved pollen data from the Eifel region in western Germany indicate an overall climatic shift around 5 ka, with lower July temperatures and higher mean annual precipitation since then^79^. These changing climatic conditions resulting in higher soil moisture since the late middle Holocene are the most likely explanation for the end of Chernozem/Phaeozem formation and the beginning of their degradation by subsequent clay relocation and thus Luvisol formation.

### Regional soil reworking rates

The presented effective soil reworking rates indicate that long-term vertical soil mixing by natural bioturbation in the Central German Chernozems/Phaeozems has rates between 0.014 to 0.051 mm/a (Fig. 7A,B), showing maxima between 20 to 30 cm. These are in the same order of magnitude as the maximum effective soil mixing rates in a Cambisol setting in Spain where bioturbation by ants presumably dominated^27^. For the Spanish Cambisols, however, these maximum rates were mainly recorded in the upper 5 cm of the soil profiles. When also considering the soil depths into our calculations, we observe approximately ten times higher effective soil mixing rates for our Central German Chernozem. Interestingly, Madsen et al.^80^ infer an order of magnitude larger vertical mixing rates of at least 6 mm/a for a lugworm dominated Wadden Sea setting in Denmark, suggesting that lugworm bioturbation is significantly more intense than the natural bioturbation in Chernozems/Phaeozems.

The uppermost sample in profile BH II at 10 cm (BHII-1) shows a sudden increase in the luminescence-based effective vertical mixing rate, being vastly larger than the effective mixing rates associated to natural bioturbation. We suggest that this high rate of ca. 0.42 mm/a is associated to anthropogenic mixing caused by local ploughing activity prior to burial mound construction. Since prehistoric ploughing during the Bronze Age exceeded depths of 15 cm only in exceptional cases^81,82^, it becomes apparent why only the top sample of BH-II was potentially affected by ploughing. The fact that no strongly increased effective soil mixing rates were observed at site BH III, located ca. 24 m to the north, could either indicate its possible location outside a prehistoric agricultural field, or surface levelling in the northern area prior to mound construction by removal of the uppermost part of the natural soil. The 13 cm shallower relative depth of the stone layer with respect to the Bronze Age surface at site BH III compared to BH II obviously favours the latter interpretation. However, similar stabilisation ages at similar depths at both sites (Fig. 6) suggest a location of the stone layer at site BH III closer to the Bronze Age surface already prior to Chernozem formation. The likewise lower depth of the Bwtb horizon in profile BH III compared to BH II (Fig. 3) could be explained by the higher density of the Saalian moraine below the stone layer compared to the sand loess, slowing down percolation processes already at lower depths of profile BH III compared to BH II.

In summary, we conclude that effective soil mixing rates in Central German Chernozems/Phaeozems are much more effective than in Spanish Cambisols, but less effective than lugworm bioturbation in the Danish Wadden Sea. Furthermore, much higher effective soil mixing rates caused by anthropogenic ploughing potentially allow to identify areas of prehistoric ploughing activity in paleosols.

## Conclusion

We applied a novel single-grain feldspar luminescence protocol to date beginning and end of the formation of a Central German Chernozem that was covered by a burial mound during the Early Bronze Age at about 3.8 ka. This buried Chernozem, which has since been protected from recent anthro-pedogenic processes, forms a unique archive of Central German Chernozems/Phaeozems, as the region has since been intensively used for agriculture resulting in strongly degraded modern soils. The results demonstrate that the applied pIRIR single-grain feldspar luminescence method is well suited to derive meaningful formation ages and process rates for Chernozems/Phaeozems. By dating the period in which bioturbation was the predominant soil-forming process, we were able for the first time to directly determine the time of the Chernozem/Phaeozem formation in Central Europe. We conclude from the luminescence-based effective soil reworking rates that vertical mixing due to earthworm bioturbation in Chernozems/Phaeozems is more intense than ant-dominated bioturbation, but significantly less intense than lugworm bioturbation in tidal flats or anthropogenic ploughing. Hence, the latter effect potentially allows to identify prehistoric ploughing activity in paleosols.

Furthermore, the single-grain luminescence data demonstrate that Chernozem/Phaeozem formation started at latest during the Early Holocene prior to Neolithic settlement. In contrast to other regions, we can therefore clearly exclude that the Central European Chernozems/Phaeozems are exclusively of anthropogenic origin. Additionally, the luminescence data indicate that Chernozem/Phaeozem formation ceased around 6–5 ka when the regional climate changed towards more humid conditions.

## Supplementary Information

Supplementary Table 1.

## Data Availability

The datasets used and/or analysed during the current study available from the corresponding author on reasonable request.
