# Supplementary material for: Deciphering timing and rates of Central German Chernozem/Phaeozem formation through high resolution single-grain luminescence dating
Source: Sci Rep. 2023 Mar 23;13:4769. doi: 10.1038/s41598-023-32005-9 (PMC10036524; doi:10.1038/s41598-023-32005-9)
Supplement: Supplementary file 1 — Supplementary Table 1. [file 41598_2023_32005_MOESM1_ESM.pdf]

Supporting Table 1: Apparent and effective soil reworking rates derived from the luminescence samples

Field ID	Lab. ID	Depth below palaeosol [m]	Age [ka]	Apparent soil reworking [mm/a]	Fraction of mixed grains [%]	Effective reworking [mm/a]
BH-II-1	NCL-1119001	0.09 ± 0.02	3.9 ± 0.6	0.90 ± 0.41	46.7	0.420 ± 0.189
BH-II-2	NCL-1119002	0.18 ± 0.02	5.3 ± 0.6	0.12 ± 0.04	39.5	0.047 ± 0.017
BH-II-3	NCL-1119003	0.28 ± 0.02	6.0 ± 0.7	0.12 ± 0.04	42.0	0.053 ± 0.018
BH-II-4	NCL-1119004	0.38 ± 0.02	6.7 ± 0.7	0.13 ± 0.04	26.9	0.035 ± 0.012
BH-II-5	NCL-1119005	0.49 ± 0.02	9.6 ± 1.1	0.08 ± 0.03	32.0	0.027 ± 0.009
BH-II-6	NCL-1119006	0.59 ± 0.02	13.1 ± 1.2	0.06 ± 0.02	44.4	0.028 ± 0.008
BH-III-1	NCL-1119007	0.08 ± 0.02	5.5 ± 0.6	0.05 ± 0.02	51.2	0.024 ± 0.011
BH-III-2	NCL-1119008	0.18 ± 0.02	5.9 ± 0.5	0.09 ± 0.03	50.0	0.043 ± 0.013
BH-III-3	NCL-1119009	0.28 ± 0.02	6.1 ± 0.7	0.12 ± 0.04	41.7	0.051 ± 0.018
BH-III-4	NCL-1119010	0.38 ± 0.02	7.1 ± 1.0	0.11 ± 0.04	23.4	0.027 ± 0.010
BH-III-5	NCL-1119011	0.48 ± 0.02	10.2 ± 1.4	0.08 ± 0.03	18.4	0.014 ± 0.005
